# The political ecology of stakeholder-driven climate change adaptation: Case study from Ntalale ward, Gwanda district, in Zimbabwe

**DOI:** 10.4102/jamba.v10i1.419

**Published:** 2018-03-27

**Authors:** Alexio Mbereko, Moses J. Chimbari, Samson Mukaratirwa

**Affiliations:** 1 College of Health Sciences, University of KwaZulu-Natal, South Africa; 2 School of Life Sciences, University of KwaZulu-Natal, Westville Campus, South Africa

## Abstract

Vulnerable rural communities face climate change-related shifts in rainfall patterns, particularly droughts and floods. The study investigated how Ntalale ward households in Gwanda district of Zimbabwe interpret climate change and adapt to its stressors in the context of the Zimbabwean political economy. Focus group discussions and interviews collected qualitative data. The community has experienced the following climate change-related risks: droughts, floods, heatwave and intra-seasonal rainfall variability. Droughts were reported to be occurring more frequent in the past 25 years as compared to the period before 1991. Ntalale area experienced floods in the 2002–2003 rainy season only. Respondents generally perceived that the rainy season had changed in the past 5 years, with the season now beginning in December and ending in March. The households have resorted to shifting cultivation practices, replanting, use of wetlands in preference to upland fields, changing of seed varieties or crops, selling of livestock and informal trading as coping strategies. Although non-governmental organisations have assisted the community to set up irrigation schemes, a few selected community members have benefited from the initiative. The Ntalale community has experienced four climate change-related risks and institutions have assisted the community. It is recommended that cooperation between households and institutions is key in developing stakeholder-driven adaptation strategies.

## Introduction

The term ‘stakeholder’ may refer to an individual, group, party, community and organisation that has potential to influence or be influenced by adaptation strategies or programmes (Vervoort et al. 2014). In sub-Saharan Africa, stakeholder groups with diverging interests find themselves responding to the impacts of climate change on socio-ecological systems (Dunford et al. 2015; Vervoort et al. 2014). The impacts of climate change are multi-scaled, stretching from the individual and national up to global level. Climate change has differential impacts amongst stakeholders across different geographical scales and thus presents an adaptation equity problem (Tol et al. 2004). Despite differences in power, vision, values and focus, the stakeholders have a common goal regarding adaptation to climate change stressors. Traditionally, linear responses to climate change impacts on socio-ecological systems were promoted, but these have become weak especially in rural African communities mainly because of heterogeneous livelihoods (Vervoort et al. 2014). The response to differential impacts of climate change on households is further weakened by some unintended outcomes of scientific innovations supported by politicians, government sectors and non-governmental organisations (NGOs) (Chambers 1994; Mbereko et al. 2015a). Thus, there is need for demand-driven adaptation strategies to climate change that will co-evolve with social actors in the political economy context (Vervoort et al. 2014). Thus, the adaptive capacity of local stakeholders at the household level is enhanced or inhibited by stakeholders from the village to the national level. Hence, this article seeks to understand household interpretation of and adaptation strategies towards climate change stressors in the context of the local political economy.

Adaptation means adjusting to ongoing and future climate changes (Heltberg, Siegel & Jorgensen 2009) and may be influenced by many factors, including the protection of economic well-being or improvement of safety (Adger, Arnell & Tompkins 2005). In order to deal with challenges of climate change at the local level we need to go beyond vulnerability analyses and focus on coping and adaptation strategies. Adaptation assumes that a catastrophic damage to the natural systems should not necessarily result in disastrous and irreversible damage to humans (Adger et al. 2005; Heltberg et al. 2009). Much of the adaptation to climate change has been reactive in the sense that it is triggered by past or current catastrophic events (Adger et al. 2005). Thus, there is the need for scientists to understand future climate change scenarios in order to develop multi-scalar anticipatory adaptation capacity to climate change perturbations. Adaptation takes place against a structural hierarchy, but the stakeholder levels interact. Individuals, communities and institutions have different reasons for adaptation. Rural people’s interest would be to benefit by deriving a livelihood (Adger et al. 2003). Government and public bodies’ interest would be to protect their citizens and advancing their political careers (Adger et al. 2005). Rather than focussing on single interventions and single adaptation actions, researchers should consider the interests of different stakeholders in order to help co-manage change along continuously adaptive pathways, and attending to shifting contextual challenges (Vervoort et al. 2014).

## Stakeholder adaptation to climate change

In recent years, climate change has not been limited to the environment and socio-economic impact science, a wide range of policy-makers and stakeholders appreciate its increasing strategic importance in national economics and politics, and global power dynamics (Adger et al. 2009; Harrison et al. 2013). The Delhi Declaration from the Eighth Conference of the Parties in November 2002 intensified stakeholder involvement through the institutionalisation of the Climate Change Adaptation Fund (CCAF) for vulnerable countries (Adger et al. 2003). What was once a purely scientific question has an overt political and economic dimension emanating from multiple actors with different interests (Klein 2009). Policy and social sciences research compliment natural sciences in understanding community adaptive capacity as they analyse the capacity of governments, civil society and markets to deal with climate-related stresses (Adger et al. 2003).

Adaptation is within a political economic context, which will advantage some stakeholders over others. Critics of the National Action Plans for Adaptation (NAPAs) argue that they have not been entirely successful as they ignore key long-term adaptation concerns like future population pressure (Hardee & Mutunga 2010). Furthermore, NAPAs in developing countries have not successfully linked to the national development processes, thus creating discord with the national development policies (Hardee & Mutunga 2010).

In the current adaptation to climate change framework, scientific knowledge has been concentrated at the global and national levels. However, the scale at which stakeholders require scientific information for adaptation is rarely at the global or national scale but rather at local scales (Holman et al. 2008). Empirical and theoretical evidence points to individuals and societal adaptations to climatic and biophysical risks throughout history (Adger et al. 2009; Heltberg et al. 2009). According to Adger et al. (2009), the resource irregularities offered by different climates and the precariousness which emerges from the vicissitudes of climate have both acted as significant stimuli throughout human history for social and technological innovation. The social actors construct new realities using innovations they value when situations change (Mbereko et al. 2015a). In the 1990s and before, farm mechanisation was the dominant discourse for enhancing rural resilience to perturbations (Chambers 1994). With globalisation, the social actors in rural areas find themselves in a wider system characterised by politics, culture shock, markets, value chains, supply systems and all the links between producers and consumers (Scoones et al. 2008). The global and national institutional oppress the rural social actor, social ecology has demonstrated how rural poor can revoke their cooperation with institutional innovations, rendering large investments from above ineffective (Mbereko et al. 2015a; Scott 2008; Vervoort et al. 2014).

There is a research gap on the political economy of grassroots climate change adaptation in the political and socio-economic context. While global and national interests aim at perfecting the policy framework, scientists need to explore the current grass roots adaptation strategies as humans have been known to adapt to climate change for a long period. Thus, there is the need to interrogate how individuals and communities are adapting and how they interact with other stakeholders who are part of the structure.

### Description of study area

**FIGURE 1:**
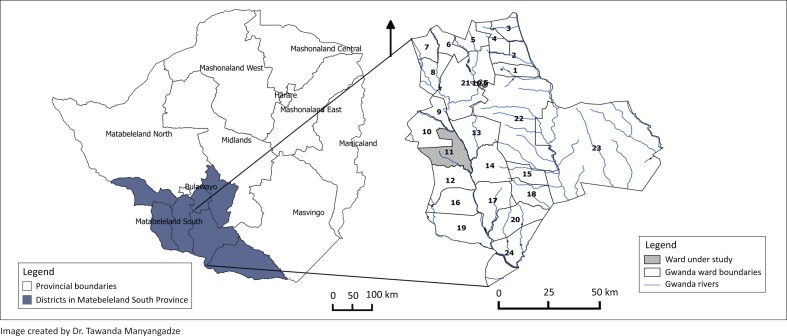
Map showing the location of Ntalale (Ward 11) in relation to Zimbabwe.

This article presents findings of a component of a bigger study on the prevalence of malaria and bilharzia in the context of climate change. The bigger study was conducted in wards 11, 15 and 18 in Gwanda rural district in Zimbabwe (Figure 1). However, Ward 11 also called Ntalale ward, was purposively sampled because it experiences more frequent droughts and floods (Figure 1). Gwanda rural district is one of the districts in Matabeleland South (Figure 1). The province is located in the south western part of Zimbabwe. Zimbabwe is divided into five natural ecological regions on the basis of rainfall patterns and topography. Gwanda lies in Region 5 found in the lowland areas of the country, lying up to 900 m above sea level and receiving annual rainfall of less than 650 mL (Macherera et al. 2016). The region is characterised by high temperatures (with an annual average of 21 ^°^C) and mid-season dry spells (Macherera et al. 2016). The main economic activities in the province include mining and livestock rearing and crop production is practiced but limited to drought tolerant crops. Ntalale is one of the 24 wards in Gwanda rural district which is semi-arid.

## Methods

The ward is composed of seven villages which were all sampled for the study. Fourteen focus group discussions (FGDs) were conducted with women and men, separately. Each FGD had less than 15 participants. Information of the research team and the requirements was sent to the village leadership so that they could mobilise FGD participants. The village leadership selected the FGD participants from their communities. The requirements set out by the researcher were as follows: participants had to be older than 18 years, mixed age groups, stayed in the area for a period of more than 10 years and household heads were preferred. The selected participants for the FGDs excluded village leadership, political leaders and no two people from the same household were to participate in the same FGD. The focus group discussions collected data on climate perturbations, coping initiatives at the community level, coping strategies at the interface of community and *ex situ* stakeholders, adaptation strategies and the role of stakeholders in adaptation.

In-depth interviews were conducted with representatives of the Gwanda Rural District Council (GRDC), one NGO and local leadership. Key informants who participated in the in-depth interviews were purposively selected. The interview participants were selected for their valued knowledge in the different aspects of climate change, local livelihoods and adaptation strategies.

Verbatim transcription of qualitative data from the FGDs and interviews was conducted by a translator. The translator was an undergraduate student fluent in the local language and English, and had experience of the study area’s culture. The translation was checked for accuracy and consistency by back and forth translations of randomly selected sections of the recordings. Community research assistants were also engaged in checking accuracy of data. The manuscripts were loaded into a computer package called Nvivo. The data were interpreted using thematic approach. The qualitative data were cleaned and contextualised in the FGD and interview settings. Data were grouped into themes and sub-themes before interpretation and linking of sub-themes was the last stage.

## Results

### Ntalale community’s experiences with climate change

Focus group discussions and in-depth interview participants said they experienced four types of climate change-related stressors: intra-seasonal rainfall variations, declining or low rainfall (locally it was known as drought), heatwaves and flooding (Table 1). Focus group discussion participants from all the villages of Ntalale indicated that the rains had become unpredictable. In the past, the rainy season started from late October to early April of the following year. The first rains were sufficient for tilling and people would wait for the second and third rains depending on the ground moisture before starting to plant crop seeds. The rains would stop for a short while in early December and farmers would take advantage of the break to weed and apply fertilisers. It was reported that they received low intensity rainfall for much of January and February, at which stage the crop would be fully grown. The crop would start to dry and be harvested after April.

**TABLE 1:** Community-perceived stressors, impacts and community responses by coping and adapting.

Climate phenomenon	Impacts	Coping strategy	Adaptation strategy
Intra-season rainfall variations	Poor germination levels	Replanting	Abandon the upland fields for wetlands and irrigated fields
			Dry planting and ridging
			Planting of drought resistant crops
	Increased labour demand	Children are removed from school to help with agricultural field work	Wealthier households employ family units as labourers Rely on social networks
	Conflicts increased from livestock destroying crops	Guard the agricultural fields	Construct fences around the fields using thorny bushes
Low rainfall	Crop failure and food insecurity	Look for assistants from social networks	Social networks
		Food handouts from donors	Donors have assisted in establishing Irrigation schemes, gardening projects and social club
		Remittances	Remittances
	Little water for livestock watering	Watering livestock at distant water source	Taking cattle to the farms
	Little pasture for livestock	Reduce the stock	-
		Rely on animal feed handouts	Feed
Flooding	Crops are destroyed	Salvage the remnants	-
		Food handouts	-
	Increased diseases (Malaria, schistosomiasis, hypertension, skin and eye infections)	Take sick people to the clinic	-
Heatwaves	Affects health	Stay under shade	-

Focus group discussion participants indicated that they have observed changes in the pattern described above since early-2000s. It was reported that intra-seasonal variations had worsened over the past 5 years. It was reported that lately the first rains come anytime between November and December. They reported these rains are usually not good enough to till the land and plant seeds. Furthermore, they said they are experiencing prolonged dry spells after the first rains. FGD participants claim that good soil moisture content is attained between December and January. The previous 2015–2016 season was reported as the worst in the past 5 years, despite reports of a normal rainfall season on the radio. One male FGD participant from Ntalale village (2016) said, ‘the people on the radio … we do not trust their knowledge of the rains and droughts, they say one thing and reality is different’. The first rains fell in late November, and they were good rains. The rains were followed by a dry spell broken by light rains towards end of December. In January, it rained heavily. Thus, cropping was delayed up to January. A dry spell followed up to March and thereafter sporadic rains fell up to end of May.

Focus group discussions participants indicated that intra-season rainfall variability impacts on the community because of poor germination, labour scheming, livestock-crop conflicts, livestock diseases, poor harvests and food insecurity. The unpredicted dry spells negatively affect seed germination. In the past the dry spells could be predicted and they usually coincided with school holidays. Under the variation scenario, the weeding season can be anytime. It was reported that in the 2015/2016 season weeding time was in January to February. It was reported that during dry spells cattle destroy neighbours’ crops and increase conflicts. The study participants said that in seasons with good rains pasture is abundant and cattle graze in the bushes. However, it was noted that in drought years cattle preferred to graze close to the roads and agricultural fields. Furthermore, the affected people met cattle that destroyed crops in drought periods with harsh response. It was perceived that cattle contracted a type of a diarrhoeal disease in those years with high variations. The dry spells negatively affected plant growth and this led to food insecurity.

**FIGURE 2:**
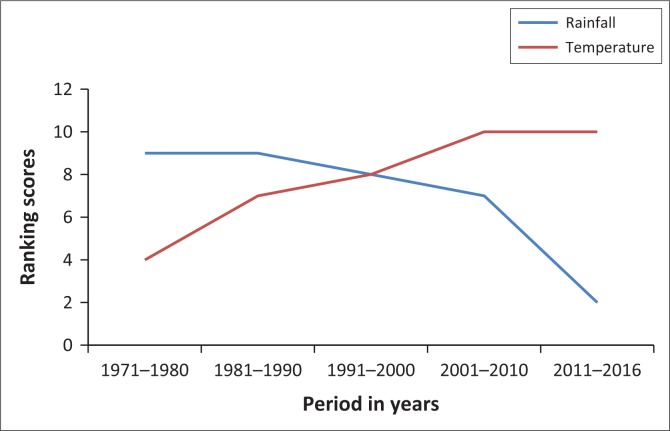
Community-perceived rainfall and temperature trends from the 10-year periods of 1971–1980 to 2011–2016.

The second climate change-related stressor experienced by the Ntalale community is drought. It was perceived that the intensity of rains declined from the period 1971–1980 to 2011–2016 (Figure 2). Temperature was perceived to be increasing (Figure 2) and droughts were reported to be more frequent within the past 25 years compared to the years before 1991. The following years were reported as drought years: 1991/1992, 2001/2002, 2007/2008, 2010/2011, 2013/2014 and 2014/2015. The major impacts of droughts reported included crop failure, little water for livestock and little pasture for livestock (Table 1). It was reported that the drought years in Ntalale are severe as the soils drain the little available water quickly. In all the FGDs they agreed that in a drought years livestock die. They said that cattle die first, followed by goats and fowl, with donkeys contributing the least mortalities.

People who participated in the study reported that the area experienced floods in the 2002–2003 agricultural season. The floods were associated with cyclone Eline that prevailed in that year. The floods damaged houses and other infrastructure. The respondents reported that floods are better than drought periods because floods raised the water table and field reservoirs, thereby making drinking water for livestock available throughout the year.

Informants reported that heatwaves are a recent phenomenon in the Ntalale area and have been noticeable in the last 5 years. They have been known to take place between October and December. Heatwaves scorch the crops and dry up the ground. It was reported that heatwaves impact human health. Communities have noticed that people with chronic high blood pressure get sick during the heatwaves. Other health problems include skin and eye sight problems.

### Coping and adaptation strategies

#### Intra-seasonal variation

In the short term when households are confronted by intra-seasonal variation of rainfall, they respond in three main ways. Firstly, it was reported that late onset of the rainy season results in poor germination as most of the farmers practice dry planting. In the event of poor germination, farmers repeatedly replant (Table 1). It was reported that the 2015–2016 season was one of the worst in recent times as farmers had to replant several times. Replanting was reported to be a problem as the farmers have to buy seed over and over again. It was reported that some poorer farmers do not get seed to replant and do not realise good harvests. Relatively wealthier farmers can afford to replant until a desired level of germination is attained. In the 2015/2016 season some farmers reported replanting three times, until a desired germination level was realised. Poor people have to borrow seed or money to buy seed to replant. In some cases people have to work for those with a lot of seed to get some as payment.

In order to deal with poor plant germination Ntalale community members resorted to planting drought tolerant seeds, abandoning uplands for wetlands and irrigated fields and dry planting (Table 1). They said people are now turning to drought tolerant crops like sorghum, water melons and drought tolerant maize. Key informant interviews claimed that the local community is not resorting to the drought tolerant crops quickly as some from other wards. People do not believe that the dry spells will continue in the future. Some think it is a problem of people abandoning Njelele shrines (rain making shrines south of Bulawayo). It was reported that households that farm under irrigation and wetlands had higher returns in the previous years which were characterised by high seasonal rainfall variability.

The Ntalale community members indicated that the rainy season had shifted and this caused labour shortages. It was alluded that in the past weeding was done in December when schools would have closed for holidays. The children would help with field activities during that period. With rainfall variability, weeding time now coincides with the first term of schools in January. As a coping mechanism the parents withdraw the children from schools to help with field work in the event of labour shortages. It was reported in FGDs that some people are starting to engage hired labour if they can afford to. Those who cannot afford it rely on their social networks, especially their extended family, friends and neighbours. Some of the people within social networks work for food or labour exchange.

It was reported that livestock and other wild animals (especially duikers) destroy crops. FGD participants perceive that livestock and animals feed in the fields because there is no forage in the bush due to poor rains and the heat. These two weather phenomena reduce forage for livestock and animals. Thus, in the long term, farmers may have to fence their fields using thorny bushes, something that used to be done for gardens only.

#### Droughts

In the years when the community experienced droughts, crop failure and food insecurity prevailed. In the event of food crises, the community reported that in order to survive they would borrow within their social networks. The neighbours who were friends were the first port of call, followed by other neighbours, then relatives and lastly, other village members. When asked whom they would not help, the unanimous answer was anyone who was known for stealing. It was reported that borrowing arrangements involving food were the prerogative of the wife. However, women indicated that husbands are now getting involved. Most men justify getting involved in food issues by saying that they are the ones who work for the food and that living conditions have become hard. One middle aged man from Ntalale (2016) said, ‘When I bring food into the house for my family. I know how many days the food should take us’. Women reported that some men harassed wives over lending out food to their friends’ household. However, notwithstanding the harassment, most women continued to lend food to their friends who were in need.

Some households rely on remittances in order to cope with and adapt to food shortage. Remittances were said to be unreliable and the amount being sent was negatively affected by exchange rates. One man said:

People overate remittances from South Africa; some of our children do not have proper jobs that pay well. These days restrictions have been put on basic food imports by the government. When they send money the exchange rate of the dollar to the rand ‘kills us’ [*they lose out*]. (One middle aged woman from Mandihongola village who participated in an FGD, 2016)

It was reported that before restrictions on food import were imposed, remittances were more helpful than now. People in the diaspora send money, but with the current exchange rates the money does not contribute much to the households’ livelihoods.

Drought periods were reported to be responsible for reducing water for livestock and poor pastures. Ntalale community responded by watering livestock at distant water sources, selling livestock and feed. Some farmers said they were selling livestock to raise money for buying stock feed to sustain the remaining heard. The stock feed will ensure the survival of the remaining heard. The other adaptation strategy was the entrustment of cattle to people in the previously white-owned cattle ranches (resettled farms). People were resettled in the farms during the fast track land reform. The ranch lands were held under private property, with cattle ranching as the dominant land use. These farms were reported to have good watering infrastructure, inherited from the white commercial farmers. The new inhabitants agreed on a payment to look after cattle from the nearby communal areas like Ntalale ward. The communal areas have been inhabited by ‘peasants’ and because of overstocking the pastures are poor and watering infrastructure scarce. It was reported that the people resettled in the ranches require cattle as payment and not money.

#### Floods

The two impacts of the flood experienced in the Ntalale ward were destruction of crops and increased ill health. The community did not report any adaptation strategies, as floods rarely occur in the area. The coping strategies used included reliance on donor food handouts. It was reported that diseases like bilharzia, malaria and diarrhoea increased during and just after the floods. The people who contracted waterborne diseases were taken to hospital for treatment.

#### Heatwaves

In Ntalale, it was reported that heatwaves had negative impacts on health. FGD participants said they observed that during periods of heatwaves, people who suffer from chronic hypertension experience increased blood pressure. Other people reported having headaches and sore eyes. People said they had limited options except to stay in the shade.

## Institutional structure

FGDs participants identified a total of 25 organisations that worked in the Ntalale ward from 2000 to 2016 and these can be categorised into four clusters: religious groups, government departments and parastatals, NGOs, and research and academic institutions (Table 2). It was reported that churches mainly helped with food, clothing and blankets in times of both drought and floods. During drought and flooding, the community received aid from the GRDC, Ministry of Health and Child Care and Ministry of Agriculture through their department of Agricultural Extension Services (AREX). The grain Marketing Board (GMB), Department of Veterinary Services and Department of Social Welfare were reported to have assisted the community during drought times. The assistance offered by the government and parastatals was within the institution’s domain mandated by central government.

**TABLE 2:** Reported institutions operating in Ntalale ward during droughts and floods and the nature of assistance.

Institutional category	Institution	Institution assists during drought periods	Institution assists during flooding periods	Assistance rendered to the Ntalale community
Religious groups	Christian Churches	√	√	Food handouts Clothing Blankets
Government departments and Parastatal(s)	Department of Social welfare	√	X	Food handouts to the vulnerable
	Ministry of Health	√	√	Medical services Health education Recommends vulnerable children for food handouts by NGOs
	Ministry of Education	X	X	Education of children on academic subjects
	Environmental Management Agency	X	X	Policing against the cutting down of trees
	Agricultural Extension Services (AREX)	√	√	Supervise gardens and irrigation agriculture Training on wetland and upstream agriculture
	The Department of Veterinary services	√	X	Training on livestock husbandry Construction of deep tanks Training on, and supervision of kraals Feed lot
	Grain Marketing Board	√	X	Food handouts
	Gwanda Rural District Council	√	√	Food sell at cheap prize Road construction
Non-governmental organisations	Moriti oa Sechaba	√	X	Dug wells and boreholes Gardening
	**Christian care**			
	Lutheran Development services	√	√	Irrigation Food distribution Construction of clinic Distribution of mosquito nets
	Pro Africa	√	X	Irrigation Agricultural practice training
	Dabani Trust	√	-	Boreholes
	Thusa nang	X	X	Supports HIV and AIDS patients
	German Help	√	X	Seed Food handouts Water harvesting training
	Australia Aid	√	√	Food hand outs Training on gardening Drainage of flooded waters
	Hand in Hand Southern Africa	**-**	X	-
	Red Cross	-	√	Temporary shelters
	C.A.R.E International	√	X	Irrigation Food handout Dug dip tanks (for goats) Money landing schemes
	World vision	√	√	Food for work Drug supply Temporary shelters
	PSI	√	√	Provided mosquito nets
	FAO	√	X	Food hand outs Constructed a warehouse
	UNICEF	√	X	Mosquito nets Financed nurses allowances
**Research**	Malaria and Bilharzia in Southern Africa (MABISA)	√	X	Information on malaria, bilharzia and climate change Training of community research assistants and community Advisory boards on bilharzia, malaria and climate change

√, assisted; X, did not assist; C.A.R.E, Cooperative for Assistance and Relief Everywhere; PSI, Population Services International; FAO, Food and Agriculture Organization; UNICEF, The United Nations Children’s Fund.

Focus group discussions respondents are of the perception that NGOs are more helpful than other institutions in times of stress. However, it was noted that some of them do not work in the area for long periods. It was reported that in some cases NGOs collaborated in their efforts to service communities and communities appreciated the outcomes of initiatives like irrigations and boreholes.

It was reported that the Malaria and Bilharzia in Southern Africa (MABISA) project was the only big research group the community had worked with since 2000. The project was said to be researching bilharzia, malaria, climate change and forestry. The research team was said to bring valuable knowledge regarding climate change and health.

**FIGURE 3:**
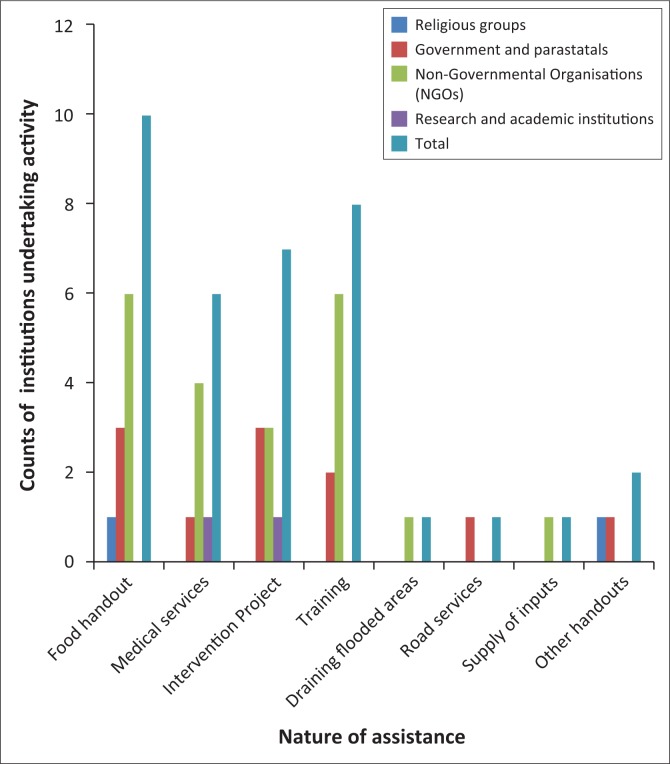
Number of institutions and the nature of assistance rendered to the Ntalale community of Gwanda Rural District, Zimbabwe.

The data from the FGDs show that the majority of organisations assisted the community by providing food handouts (Figure 3). The NGOs have provided the majority of the food handouts to the Ntalale community. More institutional support was through development projects (Figure 3). Out of the eight institutions that engaged in development projects, six were NGOs and the other two were government and parastatals. Training of local community members was the third highest activity engaged in by institutions. The training covered a number of areas including health services, parasitic disease vector identification and control, crop production, animal husbandry and water harvesting. Equal number of institutions from NGOs and government departments took part in training communities in order to build resilience to climate change-related stressors. Medical services were provided to the community by six institutions. However, NGOs had the highest number of institutions that provided health-related help. The Ministry of Health and Child Care and MABISA were the other institutions that provided health services.

**FIGURE 4:**
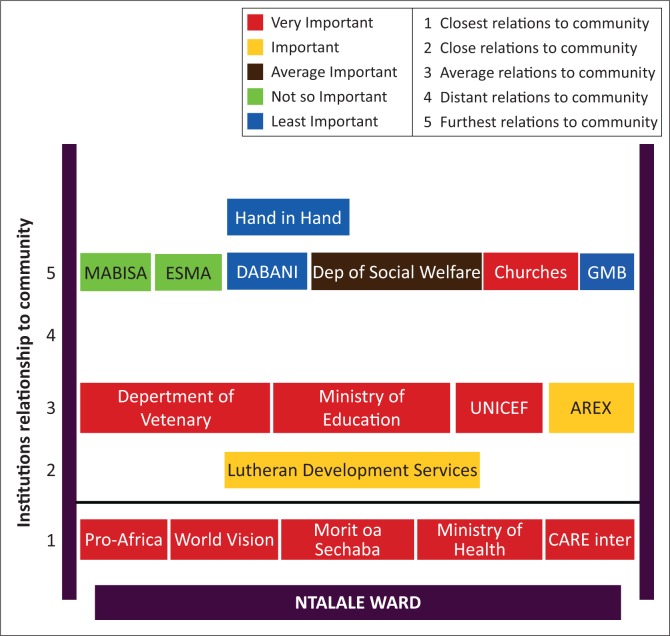
Institutions and their perceived importance during times of climate change-related stress, and social distance to the community.

The communities ranked their relationships with different institutions on a scale of 1 to 5, with 5 being the most important and 1 least important (these are represented by different shapes in Figure 4). The Ntalale study participants ranked nine institutions to be very important to their livelihoods during droughts (Figure 4). Amongst the nine are churches, three government departments and five NGOs (Figure 4). An NGO and a government department were ranked to be important to their livelihoods during drought periods as they helped vulnerable households cope. The Department of Social Welfare was ranked as averagely important to the community’s livelihoods in times of drought because their level of assistance to the community is erratic and small. MABISA and EMA were perceived not to be below averagely important. FGD participants claimed that MABISA and EMA are very new institutions and thus their assistance during drought times is still minimal. DABANI, Hand in Hand and GMB were ranked the worst. Focus group discussion participants claimed that these three institutions come once in a while and benefit very few people. Focus group discussion participants complained about the NGOs selection criteria. Participants from Ntalale ward preferred to identify their own people who should benefit using their criteria.

## Discussion

This study reports the Ntalale community’s experiences and responses to climate change in the existing institutional framework. There are a number of climatic phenomena that are being affected by climate change, but not all of them affect every part of the globe equally (Adger et al. 2003). In this study, the participants have experienced four weather conditions that are related to climate change; these are rainfall variability, poor rainy season (droughts), floods and heatwaves. A number of studies in rural Zimbabwe have focussed on rainfall variability, droughts, temperature increases and floods (Brown et al. 2012; Chikozho 2010). It is also important to note that research shows that rural communities are aware of the changing climatic conditions (Brown et al. 2012; Mtambanengwe et al. 2012; Mubaya et al. 2012). Knowledge of the changing climate affords an opportunity for rural communities to understand the impacts of climate change on their livelihoods. This study demonstrates that people from the Ntalale area are aware of climate change phenomena affecting them and their implications on their livelihoods. However, some are reluctant to develop adaptation strategies. For example, some people still grow the long-season variety crops even though they have observed that in the past 10 years the rainy season has been shorter with intra-season dry spells. The data presented here demonstrate that the impacts of changing climatic phenomena negatively affected the people. The IPCC report argues that climate change will benefit some regions and disadvantage others (Stocker et al. 2013). The findings of this study contribute to the literature that demonstrates that the southern African region is being negatively affected by climate change (Chikozho 2010; Mubaya et al. 2012).

This study demonstrates a number of coping mechanisms that were used to address short-term survival needs after a climate-related stressor struck. The coping strategies adopted to deal with the impacts of intra-seasonal rainfall variation by the Ntalale community are largely struggling as per Rugalema’s binary analysis (Rugalema 2000). These coping strategies have a medium- to long-term negative effect on the household livelihoods. For example, withdrawal of children from school to provide labour in the fields perpetuates the vulnerability and poverty cycle. On the impacts of droughts, the coping strategies adopted are mostly not erosive to the households’ livelihoods with the exception of reducing livestock numbers. By selling some of the livestock, the household reduces its asset base. The coping strategies adopted for droughts demonstrate a level of preparedness and reliance on external actors. Floods were said to not be a big problem because they bring water and it is rare to have floods in Ntalale and that it is a phenomenon that attracts immediate response from institutions. Hence, relying on donors and government is the only coping strategy used. Heatwaves are a recent phenomenon in the Ntalale area and coping strategies exist for the ill health believed to be associated with it. We have not found any medical research that links heatwaves to high blood pressure and eye problems. Other studies in Zimbabwe have focussed on the increasing temperatures rather than the heatwave (Brown et al. 2012; Mtambanengwe et al. 2012).

The Ntalale community has identified and discussed the adaptation strategies used in their ward (Table 1). The Ntalale community was avoiding dry uplands in favour of the wetlands grooves where germination and maturity chances of crops were higher. In Zimbabwe, research has demonstrated that wetlands are key farming resources and have been used over a long time by communities (Mbereko et al. 2015a). However, the Environmental Management Agency, through the *Environmental Management Act* 2002, prohibits the use of wetlands for agriculture in Zimbabwe (Mbereko et al. 2015a). The rural communities are increasingly cultivating on wetlands as a way to adapt to the frequent droughts. However, wetlands are also prone to flooding (Mbereko et al. 2015a), thus the Ntalale communities used plant phenology as an indicator of a good or bad rainy season and the decision to plough upland or wetland was based on the indigenous knowledge (Macherera et al. 2016). The alterations in planting systems were practiced by some members of the community. FGD participants complained of germination and crop failure in extreme dry conditions if one planted drought-resistant crops or practiced dry planting.

The community experiences negative effects of climate change and makes short- to long-term livelihoods adjustments. Wealthier households coped better than poorer households when germination was poor because of dry spells. Our data show that some families replanted three times in the 2015–2016 season. These are the families whose harvest was better and had more food. In contrast, poorer households could not afford the seed. Moreover, wealthier families could hire labour and could afford draught power. Furthermore, wealthier households could sell some of their livestock, as they have many, and use the proceeds to sustain human and livestock survival. Political ecologists argue that vulnerability to natural catastrophes emanates from material well-being rather than from the weather (Blaikie 1985). Drought can have differentiated livelihood outcomes for households. The study findings agree with studies that view poverty as an important determinant of environmental risk and an important constraint of adaptive capacity (Brouwer et al. 2007). The poor will be further marginalised because of lack of capital to improve their status.

Churches assisted with relief aid through handouts. Government institutions seem to be in a business-as-usual mode (Constitutional mandates). Whether in normal or crises time, government departments aim to fulfil central government mandates. NGOs are more responsive to catastrophes through relief and development programmes to enhance local resilience. Researchers come in to assess scenarios. All these are multiple actors in the Ntalale ward trying to contribute towards resilience and adaptation to climate change-related perturbations. Other studies have mapped the stakeholders that promote adaptation to climate change (Adger et al. 2005; Mtambanengwe et al. 2012). The study agrees with other studies that have demonstrated that there are physical and ecological shifts that force humans to adjust their behaviour in accordance with resource availability in order to minimise risk and vulnerability at different spatial and societal scales (Adger et al. 2005; Chikozho 2010; Dunford et al. 2015). In Ntalale, this study demonstrates the roles of community at the grassroots level; NGOs, churches and researchers at institutional level; and government and parastatals as the agents of the central government in climate change adaptation.

In Ntalale, everyone has an opportunity to rely on social networks and donors in the event of food shortage. The community has developed a high-level reliance on handouts when a catastrophe strikes. The overreliance on donor support has been blamed for causing dependency syndrome (Mutambara et al. 2014). The number of institutions rendering support to the Ntalale area demonstrates that NGOs are playing an important role. The highest number of institutions helping in the study area are offering food handouts. Data from FGDs show that during crises times NGOs provide food for them, then they implement development projects afterwards. The irrigation schemes and training are very commendable; experience from elsewhere in the country has proven them to be effective and efficient in food security (Mutambara et al. 2014).

From this study key government departments and parastatals like GMB, EMA and Social Welfare were ranked as not being helpful in times of stress to the Ntalale community. Literature from Zimbabwe has demonstrated that EMA lacks capacity to sustainably manage natural resources in rural Zimbabwe (Dube 2015; Machaka, Ganesh & Mapfumo 2013). It can be argued that EMA was ranked low because of their efforts to curb access of rural communities to key natural resources like fire wood, wetlands and streams. The Ministry of Education was perceived as not being helpful either in times of drought and floods although it carried out its mandate of managing schools in the area. This shows that some government departments are not aligning themselves to changing social pressures and continue with a ‘business as usual’ attitude. This contradicts the national and international drive for the NAPAs in Zimbabwe. Government departments are supposed to be spearheading the drive to adapt to climate change by ensuring reduction or mitigation of risk and promoting social resilience to natural catastrophe (Collier, Conway & Venables 2008; Roberts 2008). In this case, the government departments did not adequately promote adaptation to climate change. It could be partly because of the financial crises in Zimbabwe that limit government institutions from reacting to some catastrophes or engage in development projects (Mbereko, Scott & Kupika 2015b).

## Conclusion

The study contributes to climate change research through understanding of the locally constructed meanings of climate change and how communities respond to climate related stressors in the institutional settings. We conclude that the Ntalale community is mainly stressed by rainfall variability and shortage; people have adopted a number of erosive and non-erosive coping and adaptation strategies. The wealthier households have used their own initiatives to cope with and adapt to climate change. The poorer households rely on their household assets (such as household members for labour and livestock); social networks for food; NGOs for handouts and development programmes; and government for services, training and handouts. The institutions that operate within the Ntalale ward greatly influence and shape the adaptation strategies. Sustainable interventions that promote adaptation by households in this study include irrigation schemes and training. The qualitative methodology allowed for the examining of micro processes and the political theory facilitated the linking of the everyday survival struggles to the macro-institutional framework. Although Ntalale has multiple stakeholders, we contend that the household is a key stakeholder in developing adaptation strategies. It is recommended that cooperation between households and institutions is key in developing stakeholder-driven adaptation strategies.
